# Assessment of residual awareness in patients with disorders of consciousness using functional near-infrared spectroscopy–based connectivity: a pilot study

**DOI:** 10.1117/1.NPh.11.4.045013

**Published:** 2024-12-12

**Authors:** Yifang He, Nan Wang, Dongsheng Liu, Hao Peng, Shaoya Yin, Xiaosong Wang, Yong Wang, Yi Yang, Juanning Si

**Affiliations:** a Beijing Information Science and Technology University, School of Instrumentation Science and Opto-Electronics Engineering, Beijing, China; b Capital Medical University, Beijing Tiantan Hospital, Department of Neurosurgery, Beijing, China; c Chinese Academy of Medical Sciences and Peking Union Medical College, Peking Union Medical College Hospital, Department of Neurosurgery, Beijing, China; d Tianjin Medical University, Clinical College of Neurology, Neurosurgery and Neurorehabilitation, Tianjin, China; e Tianjin Huanhu Hospital, Department of Neurosurgery, Tianjin, China; f Aviation General Hospital, Department of Neurosurgery, Beijing, China; g Tianjin Huanhu Hospital, Tianjin Neurosurgical Institute, Tianjin Key Laboratory of Cerebral Vascular and Neurodegenerative Diseases, Tianjin, China; h Aviation General Hospital, Beijing, China; i Beijing Institute of Brain Disorders, Beijing, China

**Keywords:** functional near-infrared spectroscopy, resting-state, disorders of consciousness, functional connectivity, machine learning, classification

## Abstract

**Significance:**

The accurate assessment and classification of residual consciousness are crucial for optimizing therapeutic interventions in patients with disorders of consciousness (DOCs). However, there remains an absence of effective and definitive diagnostic methods for DOC in clinical practice.

**Aim:**

The primary objective was to investigate the feasibility of utilizing resting state functional near-infrared spectroscopy (rs-fNIRS) for evaluating residual consciousness. The secondary objective was to explore the distinguishing characteristics that are more effective in differentiating between the unresponsive wakefulness syndrome (UWS) and the minimally conscious state (MCS) and to identify the machine learning model that offers superior classification accuracy.

**Approach:**

We utilized rs-fNIRS to evaluate the residual consciousness in patients with DOC. Specifically, rs-fNIRS was used to construct functional brain networks, and graph theory analysis was conducted to quantify the topological differences within these brain networks between MCS and UWS. After that, two classifiers were used to distinguish MCS from UWS.

**Results:**

The graph theory results showed that the MCS group (n=8) exhibited significantly higher global efficiency ($E_{g}$) and smaller characteristic path length ($L_{p}$) than the UWS group (n=10). The functional connectivity results showed that the correlation within the left occipital cortex (L_OC) was significantly lower in the MCS group than in the UWS group. By using the indicators with significant differences as features for further classification, the accuracy for K-nearest neighbors and linear discriminant analysis classifiers was improved by 0.89 and 0.83, respectively.

**Conclusions:**

The resting state functional connectivity and graph theory analysis based on fNIRS has the potential to enhance the classification accuracy, providing valuable insights into the diagnosis of patients with DOC.

## 1 Introduction

With the continuous advancements in critical medical care and neuroimaging technologies, there has been a significant improvement in the survival rate of patients who have suffered from brain injuries, including conditions such as cerebrovascular disease, stroke, anoxia, and traumatic brain injury (TBI).^1^ Among them, a considerable number of severe cases have been diagnosed with disorders of consciousness (DOCs). DOCs are characterized by alterations in wakefulness and/or awareness,^2^ mainly including coma, defined by the complete absence of wakefulness and awareness; unresponsive wakefulness syndrome (UWS), previously known as vegetative state (VS), which is characterized by the presence of wakefulness but without awareness of themselves and the external environment; and minimally conscious state (MCS), characterized by minimal, inconsistent, yet reproducible signs of awareness.^1^^,^^3^ The accurate and prompt diagnosis of DOC is imperative for patients as it directly influences the design of individualized therapeutic strategies, the determination of prognostic outcomes, and even the decision-making process regarding end-of-life care.^4^

To date, clinical behavioral examination using the Coma Recovery Scale-Revised (CRS-R) is the standard approach for clinicians to assess the level of consciousness in patients with DOC.^5^ However, such examination is subjective and vulnerable to errors, with a misdiagnosis rate estimated to be ∼40%.^6^ In fact, the accurate evaluation of consciousness is significantly challenging due to the potential cognitive, motor, visual, and auditory deficits in those unique populations of patients, which can inevitably contribute to the risk of misdiagnosis. In addition, the fluctuating levels of consciousness, along with patient fatigue and pain, present substantial challenges in maintaining a stable condition throughout the assessment period for patients with DOC. Finally, it is crucial to consider the consistency of CRS-R scoring across different evaluators, and its test–retest reliability cannot be overlooked.^7^ Therefore, there is a pressing need for more accurate and objective methods to evaluate the residual consciousness in patients with DOC.

In recent years, significant strides in neuroimaging techniques have greatly facilitated research advancements in the field of DOC.^8^ Several studies based on functional magnetic resonance imaging (fMRI) have been conducted to detect residual awareness, establish prognostic indicators, evaluate the effects of therapeutic interventions, and explore the underlying mechanism of DOC.^9^^–^^12^ A notable example is an fMRI study by Owen et al.,^13^ who utilized a tennis-playing mental imagery task to detect the residual consciousness. In this study, the hemodynamics responses of a 23-year-old patient, clinically diagnosed with UWS, were found to be remarkably similar to those of healthy volunteers when performing the same task within the MRI scanner, indicating the presence of awareness.^13^ Subsequent studies have demonstrated that nearly 20% of patients who appear behaviorally unresponsive exhibit clear brain activation in response to specific active cognitive tasks.^14^^,^^15^ These significant findings have contributed to the establishment of a novel diagnostic category known as covert consciousness, which refers to a state of cognitive motor dissociation.^16^ In addition, researchers have further explored the differential patterns of brain activity between the UWS and MCS using fMRI under various experimental paradigms.^17^^–^^19^ Although previous fMRI studies have provided valuable information in the field of DOC, the bulky size and substantial weight of the scanner, along with its high cost, susceptibility to motor artifacts, and interference from metal implants, have collectively limited the extensive application of fMRI in longitudinal bedside monitoring of patients with DOC.

Alternatively, functional near-infrared spectroscopy (fNIRS) is a non-invasive optical neuroimaging technology that evaluates brain functional activity by measuring concentration changes of oxygenated-(HbO), deoxygenated-(HbR), and total-(HbT) hemoglobin.^20^^,^^21^ Compared with fMRI, fNIRS offers superior temporal resolution, lower cost, and enhanced portability. In addition, fNIRS is less susceptible to head movements and metal implants, making it valuable for longitudinal bedside follow-up measurements of hemodynamics with enhanced “ecological validity.”^22^ These strengths render fNIRS a promising tool for evaluating residual consciousness in patients with DOC. In recent years, accumulating fNIRS studies have been conducted to investigate the brain activity of patients with DOC in terms of the detection of residual consciousness^23^^–^^27^ and the evaluation of therapeutic effects of neuromodulation techniques.^28^^,^^29^ Specifically, the experimental paradigms for residual consciousness detection mainly include passive paradigms (e.g., auditory stimuli^30^) and active paradigms [e.g., motor imagery tasks,^5^^,^^23^^,^^31^ mental arithmetic tasks,^24^ and subject’s own name (SON) tasks^26^]. These paradigms are capable of yielding valuable diagnostic and predictive information for the assessment of the brain activity of DOC.^20^ For instance, Kempny et al.^31^ utilized fNIRS-based motor imagery tasks to evaluate the brain functional activity of patients with DOC and found that the distribution of hemodynamics of the MCS group exhibited a similar pattern to that of healthy controls. Abdalmalak et al.^20^ highlighted the potential of using the fNIRS-based motor imagery paradigm as a communication tool for patients with locked-in syndrome.^25^ Si et al.^23^ further investigated the differences in hemodynamic responses between MCS and UWS patients using active command-driven MI tasks. Apart from the motor imagery task, fNIRS-based mental arithmetic tasks^27^ and SON tasks^26^ have also been reported as effective methods for evaluating residual consciousness in patients with DOC.^24^ However, active tasks typically require patients to understand and follow the researcher’s oral commands, as well as to perform the assigned tasks. Consequently, these approaches may prove ineffective in cases where patients exhibit cognitive or physical function impairments, such as limitations in movement, attention, and memory.^27^

Unlike the active paradigm, the resting state fNIRS (rs-fNIRS) is a convenient, task-free paradigm that does not require active participation from the patient. This method is particularly valuable for exploring spontaneous fluctuations and investigating the intrinsic functional connectivity within the brain. It provides valuable insights into the baseline state of the brain function without the influence of external stimuli and the variability in the performance of patients due to differences in motor or cognitive ability.^11^ Therefore, rs-fNIRS is useful for evaluating residual consciousness in patients with DOC. By constructing the whole brain as a network, previous rs-fNIRS studies^30^^,^^32^ have demonstrated the potential of rs-fNIRS in investigating the brain’s functional activity, particularly focusing on the topological characteristics of brain networks in patients with DOC. For instance, Liu et al.^32^ found that both MCS and UWS patients showed a reduced clustering coefficient ($C_{p}$) and lower network information interaction efficiency, including global efficiency ($E_{g}$) and local efficiency ($E_{\mathrm{loc}}$), along with longer characteristic path length ($L_{p}$) compared with the healthy controls. These findings indicate that the patients with DOC exhibited impaired global connections and decreased network complexity, particularly in the prefrontal cortex (PFC). The neuromodulation effects of deep brain stimulation (DBS) on patients with DOC were investigated using the rs-fNIRS technique.^33^ The results showed that global communication efficiency ($E_{g}$) values were significantly correlated with CRS-R scores, indicating that global communication efficiency can be used as a promising biomarker for DOC recovery.^30^ Although the previous studies have yielded valuable insights into brain functional activity research within the field of DOC, the number of rs-fNIRS studies remains fragmented and limited. The reliability of the rs-fNIRS paradigm for the detection of residual consciousness in patients with DOC needs further investigation. In addition, a more profound exploration is needed to understand the differences in brain networks between the MCS and UWS groups.

In view of this, resting state fNIRS data were acquired from the prefrontal, motor, and occipital cortices of patients with DOC. The research goals of the current study were as follows: The primary aim was to test the feasibility of utilizing rs-fNIRS for evaluating residual consciousness. The secondary aim was to explore the distinguishing characteristics that are more effective in differentiating between the UWS and MCS and to identify the machine learning model that offers superior performance for classification.

## 2 Materials and Methods

### 2.1 Participants

In this study, 18 patients (15 males and 3 females) were recruited from the Aviation General Hospital. Inclusion criteria include (1) etiology of TBI, viral encephalitis (VE), stroke, hypoxic ischemic encephalopathy (HIE), meningoencephalitis, etc.,^34^ with a duration of more than 28 days and in a stable condition; (2) diagnosis as UWS and MCS according to the CRS-R. The CRS-R consists of six subscales designed to evaluate auditory, visual, motor, oromotor, communication, and arousal functions, which are summed together to yield a total score with a possible range of 0 to 23.^23^ Clinical diagnosis criteria^35^ for MCS and UWS are illustrated in Table 1. CRS-R scoring requires that the patient’s vital signs are normal and that the patient’s scoring has been performed at least three times within 1 week before enrollment. Exclusion criteria include (1) history of epilepsy or psychiatric or neurological disorders, (2) long-term use of sedative or antiepileptic drugs, (3) uncontrollable infections or other serious medical diseases, and (4) inability to obtain informed consent from the legal caregivers.^23^

**Table 1:** CRS-R criteria for MCS and VS.^35^

CRS-R scales	MCS	VS
Auditory	3 to 4 or	≤2 and
Visual	2 to 5 or	≤1 and
Motor	3 to 6 or	≤2 and
Oromotor	3 or	≤2 and
Communication	1 to 3	0

CRS-R, coma recovery scale-revised; MCS, minimally conscious state; VS, vegetative state.

In this study, written informed consent for each subject was obtained from the patient’s legal guardians. The experimental protocol of this study was approved by the ethics committee of the Aviation General Hospital. The clinical characteristics of the DOC patients are shown in Table 2.

**Table 2:** Clinical characteristics of patients with disorders of consciousness.

No	Diagnosis	Age	Gender	CRS-R	Duration of DOC/days	Etiology	Site of injury
1	MCS	69	M	9(132102)	99	TBI	Supratentorial cerebral cortex
2	MCS	69	M	10(232102)	154	TBI	Supratentorial cerebral cortex
3	MCS	61	M	9(132102)	315	TBI	Supratentorial cerebral cortex
4	MCS	19	M	9(132102)	182	VE	Cerebral cortex
5	MCS	51	M	10(232102)	104	Stroke	Right side supratentorial thalamus
6	MCS	8	M	15(244203)	162	HIE	Supratentorial thalamus
7	MCS	67	F	10(133102)	78	TBI	Supratentorial cerebral cortex
8	MCS	67	F	12(333102)	113	TBI	Supratentorial cerebral cortex
9	UWS	62	M	5(002102)	52	Stroke	Supratentorial cerebral cortex
10	UWS	31	M	7(112102)	133	TBI	Supratentorial thalamus
11	UWS	52	F	8(122102)	152	Stroke	Supratentorial cerebral cortex
12	UWS	69	M	8(122102)	422	Stroke	Right side supratentorial thalamus
13	UWS	50	F	7(112102)	451	Stroke	Left side supratentorial thalamus
14	UWS	61	M	8(122102)	314	TBI	Supratentorial cerebral cortex
15	UWS	72	F	5(112100)	95	HIE	Cerebral cortex
16	UWS	55	M	7(112102)	198	HIE	Cerebral cortex
17	UWS	50	F	8(122102)	66	TBI	Supratentorial cerebral cortex
18	UWS	65	F	7(112102)	314	Stroke	Supratentorial thalamus

CRS-R, coma recovery scale-revised; DOC, disorders of consciousness; MCS, minimally conscious state; UWS, unresponsive wakefulness syndrome; M, male; F, female; TBI, traumatic brain injury; VE, virus encephalitis; hypoxic ischemic encephalopathy (HIE).

### 2.2 Experimental Design

In this pilot study, we employed fNIRS technology to measure the resting state data from the patients with DOC. By constructing functional connectivity and applying graph theory analysis, we extracted the characteristics that could reflect the differences between the MCS and UWS groups. Subsequently, two classifiers were used to distinguish between MCS and UWS. The main research goals were twofold: on the one hand, to further determine the viability of using rs-fNIRS for assessing residual awareness; on the other hand, to investigate the distinguishing features that are more effective in differentiating between the UWS and MCS, and to determine the machine learning model that yields superior performance for classification.

### 2.3 Data Acquisition

**Fig. 1:**
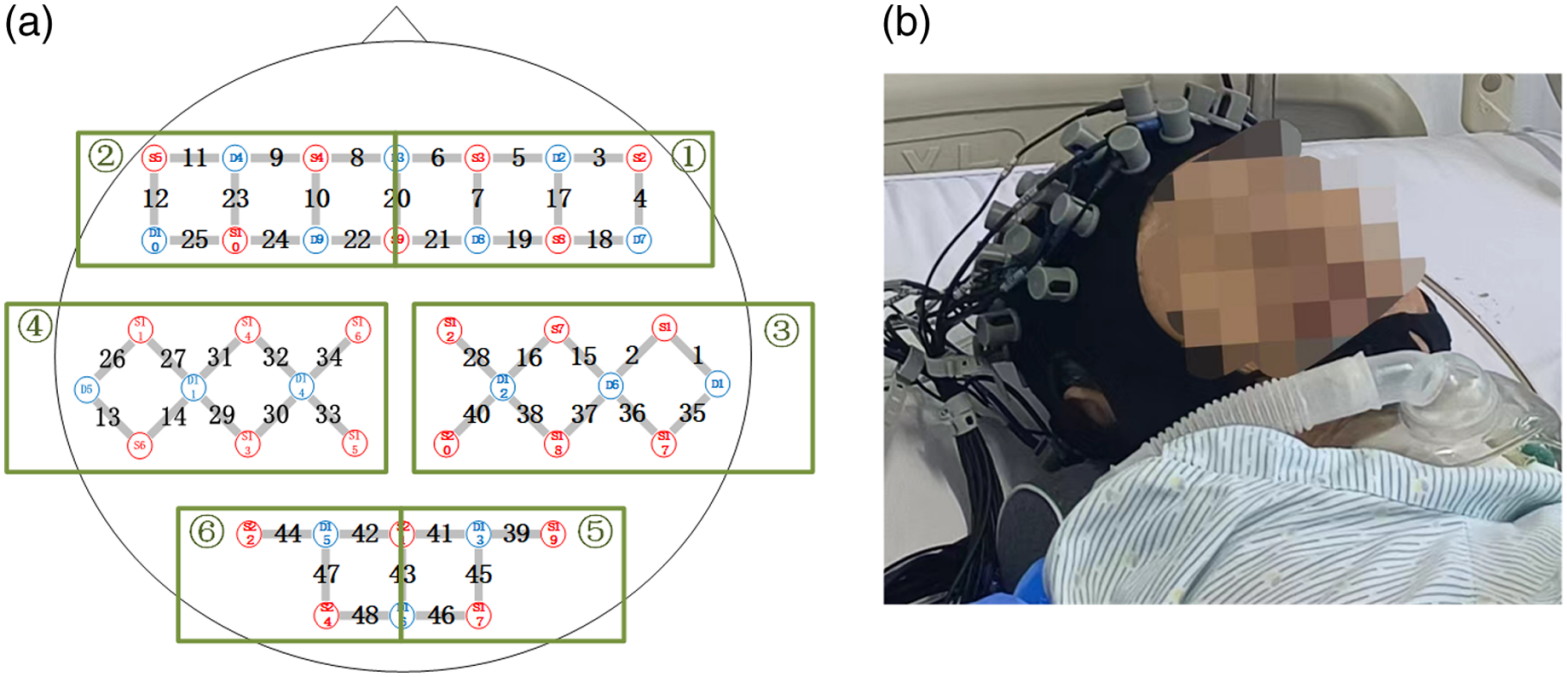
Configuration of the experiment. (a) Arrangement of fNIRS optodes on the head. ① R_PFC (right prefrontal cortex), ② L_PFC (left prefrontal cortex), ③ R_MC (right motor cortex), ④ L_MC (left motor cortex), ⑤R_OC (right occipital cortex), ⑥ L_OC (left occipital cortex). (b) Photograph of the experimental setup.

Resting-state fNIRS data were acquired using the NirSmartII-3000A near-infrared brain imaging system (Danyang Huichuang Medical Equipment Co., Ltd., Jiangsu, China). Two wavelengths, 730 and 850 nm, were used to detect the concentration changes in HbO, HbR, and HbT of the brain in real time. The fNIRS system consisted of 24 sources and 16 detectors, totally yielding 48 optical channels, with 3-cm source-detector separation. The arrangement of fNIRS optodes was based on the 10 to 20 international standard electroencephalography (EEG) system. Specifically, the 48 optical channels were symmetrically positioned over the areas of R_PFC (right PFC, channels 3, 4, 5, 6, 7, 17, 18, 19, 20, and 21), L_PFC (left PFC, channels 8, 9, 10, 11, 12, 20, 22, 23, 24, and 25), R_MC (right motor cortex, channels 1, 2, 15, 16, 28, 35, 36, 37, 38, and 40), L_MC (left motor cortex, channels 13, 14, 26, 27, 29, 30, 31, 32, 33, and 34), R_OC (right occipital cortex, channels 39, 41, 43, 45, and 46), and L_OC (left occipital cortex, channels 42, 43, 44, 47, and 48). The experimental configuration is shown in Fig. 1. The red and blue circles represent the light sources and detectors, respectively, whereas the grey connecting lines marked with numbers indicate the optical channels. The sampling rate of the fNIRS system was 11 Hz.

The acquisition period for the rs-fNIRS data was sustained for 20 min. During the fNIRS recording, the participants were lying in a comfortable position in a quiet room with normal lighting conditions. They were instructed to maintain a state of relaxation and awake and to minimize body and head movements.

## 3 Data Analysis

### 3.1 Data Preprocessing

The fNIRS data were analyzed using MATLAB 2019a (MathWorks Inc., Natick, Massachusetts, United States) and the FC-NIRS package (Xu et al., 2015, https://www.nitrc.org/projects/fcnirs/).^36^

First, the raw signal was converted into the relative changes in the concentrations of HbO, HbR, and HbT according to the modified Beer–Lambert law.^37^ After that, a band-pass filter (0.01 to 0.1 Hz) was performed to eliminate the task-irrelevant noise signals, such as heartbeats (0.8 to 1.6 Hz), respiration (0.2 to 0.6 Hz), and blood pressure fluctuations (around 0.1 Hz).^38^ Next, motion artifacts were identified and corrected using principal component analysis (PCA). The data with large and sudden motion artifacts were removed. Finally, 5-min stable hemoglobin time series were extracted for further quantitative analysis.

Importantly, to ensure high-quality fNIRS data for functional connectivity (FC) calculation and network analysis, rigorous quality control was conducted for channel pruning, based on the signal-to-noise ratio (SNR), between-channel correlation matrix, and frequency spectrum.^36^ Specifically, a channel was considered a bad channel if it met any of the following conditions:^39^ (1) The correlation coefficient with other channels was 0 or less; (2) The channel with an SNR below the threshold of 2;^40^ (3) The channel without cardiac component (∼1  Hz). In this study, further analysis predominantly focused on the HbT signal, which is derived from the sum of HbO and HbR and represents the changes in regional cerebral blood volume.^41^

### 3.2 Functional Connectivity Calculation

Each channel was defined as a node, and the whole-brain functional connectivity matrix of each participant was obtained by calculating the Pearson correlation coefficients between the time series of every pair of nodes. This procedure generated a 48×48 correlation matrix for each participant. The Pearson correlation coefficients r>0.35 within the two groups were visually represented to offer an integrated view of the functional connectivity profiles in patients with UWS and MCS, utilizing the BrainNet Viewer toolbox (https://www.nitrc.org/projects/bnv/).^42^

For further quantitative analysis, the 48 optical channels were categorized into six distinct brain regions, R_PFC, L_PFC, R_MC, L_MC, R_OC, and L_OC. The correlation values within each region were averaged to obtain the mean correlation for that region. Then, these mean correlation values were used for comparative analysis among different groups.

### 3.3 Brain Network Analysis Based on Graph Theory

Graph theory analysis was conducted to investigate the topological characteristics of brain networks in patients with DOC. The parameters of topological network efficiency have been frequently employed to characterize the capability for parallel information processing inherent within brain networks.^43^^,^^44^ The sparsity-based thresholding method ensured that the hemispheric networks in each group maintained the same number of edges.^45^ Therefore, a threshold range of 0.05 to 0.80 for the sparsity, with a step of 0.05, was selected for calculating topological network properties under each sparsity. After that, the values of the area under the curve (AUC) were calculated and used for further statistical analysis.^45^

In this study, the four global network features are characteristic path length ($L_{p}$), clustering coefficient ($C_{p}$), global efficiency ($E_{g}$), and local efficiency ($E_{\mathrm{loc}}$). Among them, $C_{p}$ and $E_{\mathrm{loc}}$ evaluate the aggregation of the network, whereas $L_{p}$ and $E_{g}$ evaluate the information transmission efficiency of the network. The definitions and formulas for these network metrics are as follows:

a. The characteristic path length ($L_{p}$) is the characteristic path length between all pairs of nodes in the network G^32^
  $$L_{p}=\frac{1}{N(N-1)}\underset{i\neq j\in G}{\sum }d_{ij},$$
  where N denotes the number of nodes in the network G and $d_{ij}$ denotes the distance between nodes i and j.
b The clustering coefficient ($C_{p}$) reflects the degree of collectivization in the network^32^
  $$C_{p}=\frac{1}{N}\underset{i\in G}{\sum }\frac{E_{i}}{D_{i}(D_{i}-1)/2},$$
  where $D_{i}$ denotes the number of edges connected to node i; where $E_{i}$ denotes the total number of edges in the subgraph, including the neighboring edges of node i.
c. Global efficiency ($E_{g}$) quantifies the ability of the network to transfer information globally^45^
  $$E_{g}=\frac{1}{N(N-1)}\underset{i\neq j\in G}{\sum }\frac{1}{d_{ij}},$$
  where $d_{ij}$ denotes the shortest path length distance between nodes i and j.
d. The average of the local efficiencies ($E_{\mathrm{loc}}$) of all nodes is known as the local efficiency^45^
  $$E_{\mathrm{loc}}=\frac{1}{N}\underset{i\in G}{\sum }E_{g}(i),$$
  where $E_{g}(i)$ denotes the global efficiency of the network $G_{i}$.

### 3.4 Classification Algorithms

To assess the potential of using resting-state fNIRS data for categorizing patients with DOC, K-nearest neighbors (KNN) and linear discriminant analysis (LDA) algorithms were used to distinguish between the UWS and MCS groups.

KNN is a non-parametric machine learning algorithm that operates on the principle of feature similarity, where the response for a data point is determined based on the “K” nearest neighbors in the feature space.^46^ In the KNN classifier, the value of K was set to 3, and the distance metric used was Euclidean. LDA serves as an effective machine learning algorithm for classification, which mainly finds the linear combination of features that can separate data representing two or more groups.^47^ The input vectors for the classifiers consisted of a combination of different features extracted from resting-state fNIRS data, whereas the output vectors corresponded to the distinct categories of DOC, that is, UWS or MCS. In this study, the performance of the classifiers was evaluated with accuracy, sensitivity, and specificity based on the confusion matrix. In addition, the classification processes were conducted with leave-one-out cross-validation.

### 3.5 Statistical Analysis

In this study, two-sample t-tests were conducted to compare the differences in correlation values and topological network properties between the MCS and UWS groups. The differences were considered statistically significant when p<0.05. In addition, the Cohen’s d values were calculated to evaluate the effect size. All the data were presented in the figures as mean values with standard errors, unless otherwise mentioned.

## 4 Results

### 4.1 Differences in Brain Functional Connectivity

**Fig. 2:**
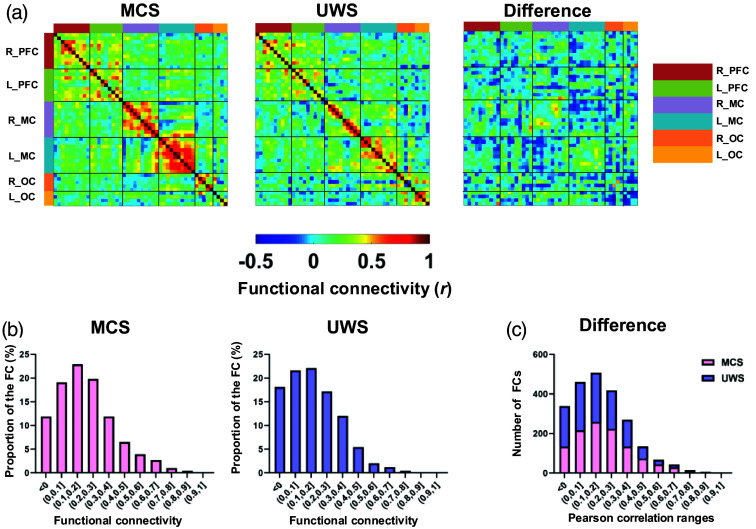
Spatial patterns of the functional connectivity in MCS and UWS groups. (a) Functional connectivity maps for these two groups and the difference in functional connectivity between MCS and UWS groups. (b) Histograms of the functional connectivity distribution for MCS and UWS groups. (c) The stacked bar chart of functional connectivity across different thresholds.

The group-averaged whole-brain functional connectivity for MCS and UWS patients is shown in Fig. 2. The warmer color indicates stronger connectivity strength, whereas the colder color indicates weaker connectivity strength. It can be observed that the functional connectivity of the MCS group was relatively higher than that of the UWS group although the spatial patterns between two groups showed obvious similarity. Quantitatively, the averaged connectivity strength across the whole brain in the MCS (0.22±0.03) group was stronger than that in the UWS (0.18±0.03) group, but the difference was not statistically significant. Furthermore, the number of functional connectivity strengths lower than 0.1 was much larger in the UWS group compared with that in the MCS group Fig. 2(c). However, there were no significant differences.

**Fig. 3:**
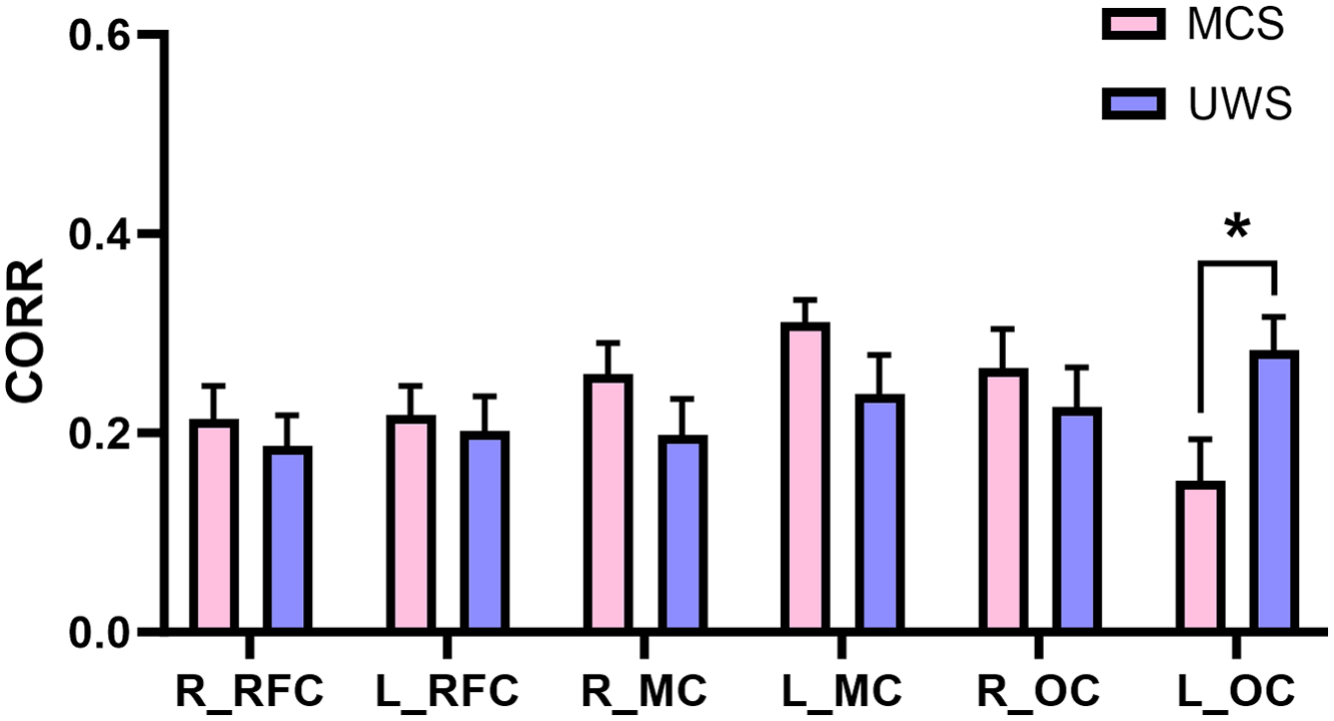
Comparison of functional connectivity strength in different brain areas between the MCS and UWS groups. *p<0.05.

The comparison of functional connectivity strength in six different brain areas between the MCS and UWS groups is shown in Fig. 3. The results revealed that the functional connectivity strength within the L_OC in the UWS group was significantly higher than that in the MCS group (p=0.0262, Cohen’s d=1.1618). However, no significant differences were observed in the functional connectivity strength within the remaining brain regions between the two groups.

**Fig. 4:**
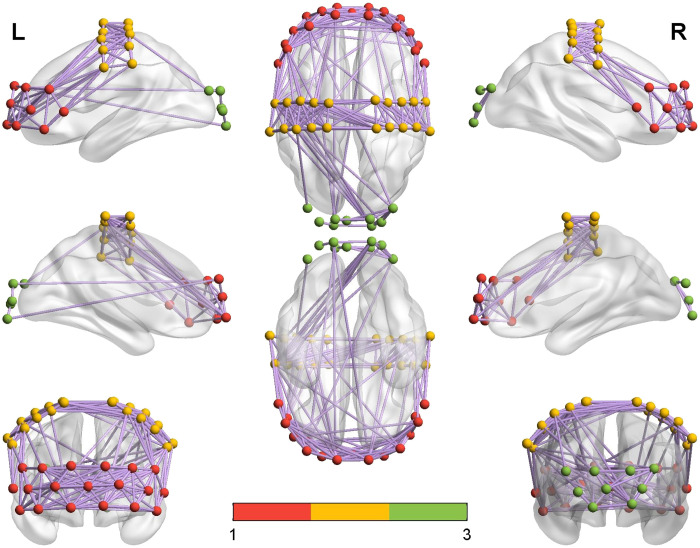
Channel connections for the MCS group with group-averaged correlation values above 0.35 (in purple). Red, yellow, and green dots indicate the fNIRS optodes in the PFC, motor cortex, and occipital cortex, respectively.

**Fig. 5:**
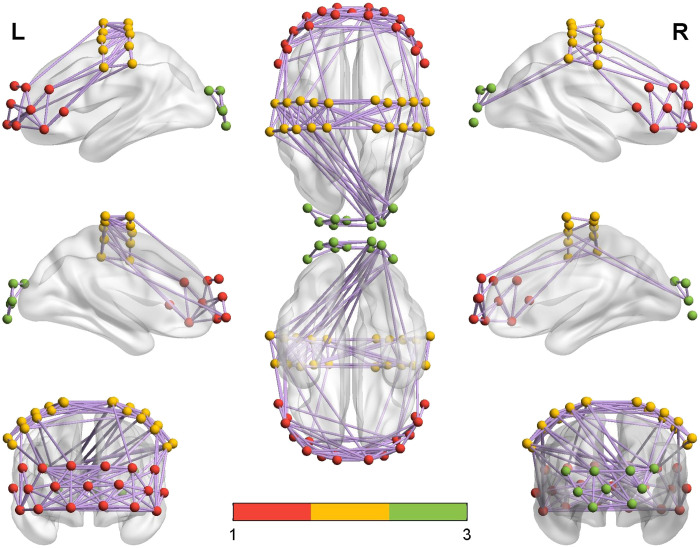
Channel connections for the UWS group with group-averaged correlation values above 0.35 (in purple). Red, yellow, and green dots indicate the fNIRS optodes in the PFC, motor cortex, and occipital cortex, respectively.

The functional brain networks for the MCS and UWS groups were visually displayed with group-averaged correlation values above 0.35,^32^ as shown in Figs. 4 and 5. It can be seen that there were more connections between PFC and motor cortex in the MCS group than in the UWS group. In contrast, there were more connections between the motor cortex and occipital cortex in the UWS than in the MCS group. However, the connections among different brain regions were not significantly different between the two groups.

### 4.2 Differences in Topological Network Properties

**Fig. 6:**
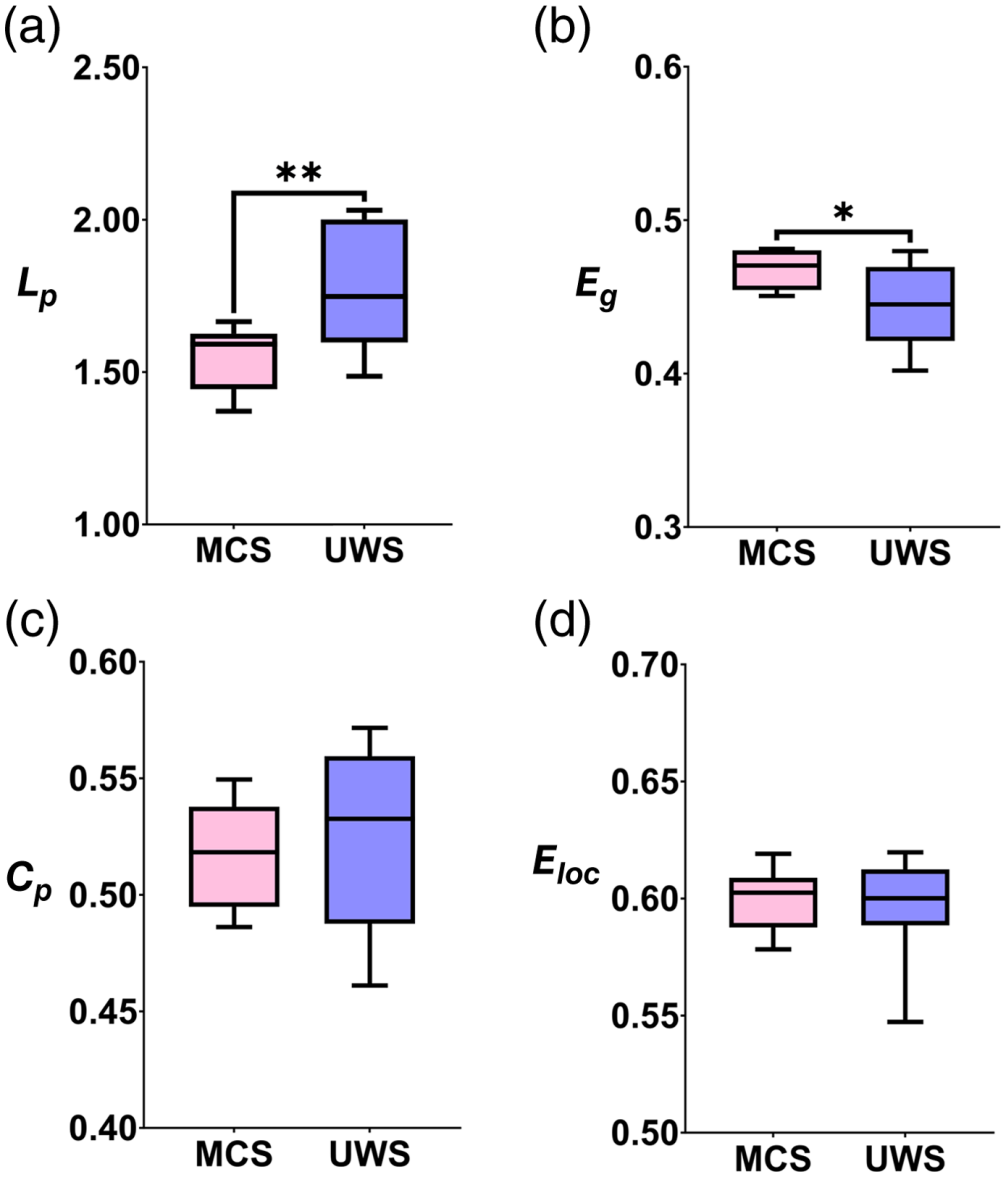
Comparisons of global network features between the MCS and UWS groups. The following four parameters were presented as properties of the global brain network in a resting state: (a) Characteristic path length ($L_{p}$), (b) global efficiency ($E_{g}$), (c) clustering coefficient ($C_{p}$), and (d) local efficiency ($E_{\mathrm{loc}}$). The error bars indicate the standard errors of the mean. *p<0.05, **p<0.01.

Quantitatively, the differences in topological network characteristics of the brain network between the MCS and UWS groups were compared. The global and local network characteristics were calculated using an integrated threshold (i.e., the AUC, including sparsity from 0.05 to 0.80 with a step of 0.05). As illustrated in Fig. 6, for global properties, the global efficiency ($E_{g}$) of the MCS group was significantly higher compared with that of the UWS group (p=0.0296, Cohen’s d=1.1326). In addition, the characteristic path length ($L_{p}$) of the MCS was significantly shorter than that of the UWS group (p=0.0097, Cohen’s d=1.3929). However, no significant differences were observed in the clustering coefficient ($C_{p}$) (p=0.5477, Cohen’s d=0.2914) and local efficiency ($E_{\mathrm{loc}}$) (p=0.7546, Cohen’s d=0.1508) between the MCS and UWS groups.

### 4.3 Classification Results

In this study, KNN and LDA classifiers were utilized to evaluate the classification performance of the MCS and UWS groups. To further improve the precision and reliability of the classifiers, the final classification accuracy was obtained by averaging the results from 20 computations. The classification results of the two classifiers with different features are shown in Table 3. Overall, when graph theory indicators ($E_{g}$, $E_{\mathrm{loc}}$, $C_{p}$, and $L_{p}$) were utilized as the feature set, the KNN and LDA classifiers achieved an ACC of 0.72, whereas the LDA classifier attained an accuracy of 0.61. When using correlation values from specific brain regions (R_PFC, L_PFC, R_MC, L_MC, R_OC, L_OC) as the feature set, both the KNN and LDA classifiers achieved an ACC of 0.67. Importantly, the hybrid feature set (i.e., the combination of graph theory indicators and correlation values) yielded superior performance, with an ACC of 0.78 for KNN and an ACC of 0.73 for LDA.

**Table 3:** Classification accuracy of the KNN and LDA with different features.

Features	KNN	LDA
GT ($E_{g}$, $E_{\mathrm{loc}}$, $L_{p}$, $C_{p}$)	0.72	0.61
Corr (R_PFC, L_PFC, R_MC, L_MC, R_OC, L_OC)	0.67	0.67
GT ($E_{g}$, $E_{\mathrm{loc}}$, $L_{p}$, $C_{p}$) and Corr (R_PFC, L_PFC, R_MC, L_MC, R_OC, L_OC)	0.78	0.73
GT $\left(E_{g},L_{p}\right)$	0.78	0.67
Corr (L_OC)	0.72	0.83
GT $\left(E_{g},L_{p}\right)$ and Corr (L_OC)	0.89	0.83

GT, graph theory; Corr, correlation value; KNN, K-nearest neighbors; LDA, linear discriminant analysis; $E_{g}$, global efficiency; $E_{\mathrm{loc}}$, local efficiency; $L_{p}$, characteristic path length; $C_{p}$, clustering coefficient; Corr, Correlation; R_PFC, right prefrontal cortex; L_PFC, left prefrontal cortex; R_MC, right motor cortex; L_MC, left motor cortex; R_OC, right occipital cortex; L_OC, left occipital cortex.

Apart from the assessment of the performance of two classifiers, we also attempted to explore the optimal features and key regions of interest (ROIs) that are predominantly associated with the level of consciousness. The performance of the feature sets with the parameters that demonstrated significant differences between the two groups was further investigated. Specifically, when using graph theory indicators ($E_{g}$ and $L_{p}$) as the feature set, the KNN classifier obtained an improved ACC of 0.78, whereas the LDA classifier got an ACC of 0.67. Similarly, when using the correlation value (L_OC) as the feature set, the KNN classifier realized an improved ACC of 0.72, and the LDA classifier reached an enhanced ACC of 0.83. Notably, when graph theory indicators ($E_{g}$ and $L_{p}$) and correlation value (L_OC) were used as the hybrid feature set, the two classifiers exhibited the best performance (accuracy = 0.89, sensitivity =0.75, specificity = 1 for KNN, accuracy = 0.83, sensitivity = 0.88, specificity = 0.80 for LDA), outperforming the other feature sets.

## 5 Discussion and Conclusion

Accurate evaluation of the level of consciousness is essential for the clinical diagnosis and management of patients with DOC. In the current study, brain network analysis and machine learning algorithms were utilized based on the resting state fNIRS to evaluate the residual consciousness and to differentiate between the MCS and UWS groups.

### 5.1 Hybrid Multivariate Feature Set Provided Better Performance

The classification results of the two classifiers with different feature sets are illustrated in Table 3. Overall, the hybrid multivariate feature set offered enhanced classification performance. Specifically, the hybrid multivariate feature set (including both the graph theory indicators and correlation values) demonstrated superior performance compared with the unimodal feature sets that relied solely on either the graph theory indicators or correlation values alone. The improved performance can be attributed to the fact that a larger number of features can provide a more comprehensive representation of brain activity, potentially capturing more information that is associated with the level of consciousness of patients with DOC. This suggests that the integration of diverse features from various sources or types can be particularly beneficial for the classification.

### 5.2 Proper Selection of Features Improved the Classification Accuracy

Identifying and selecting the features that capture the essential characteristics of the different groups is crucial for achieving higher accuracy in classification tasks.^21^ Therefore, in this study, we also endeavored to explore the classification features and ROI that are predominantly associated with the level of consciousness. Specifically, several feature sets with different kinds of characteristics were compared. Notably, the hybrid multivariate feature set, including the graph theory indicators ($E_{g}$, $L_{p}$) and the functional connectivity value (L_OC), provided the best performance (accuracy = 0.89, sensitivity =0.75, specificity = 1 for KNN, and accuracy = 0.83, sensitivity = 0.88, specificity = 0.80 for LDA). The findings indicated that global efficiency ($E_{g}$), characteristic path length ($L_{p}$), and the functional connectivity in the left occipital cortex (L_OC) were significant contributors to the classification of patients with DOC, at least in this pilot rs-fNIRS study. A recent EEG study indicated that the combination of discriminative and interpretable markers, along with automatic machine learning algorithms, is effective for differential diagnosis in patients with DOC.^48^ The LDA classifier was then applied to predict clinical outcomes 6 months after the injury, achieving a classification accuracy of 0.83. Another similar study constructed a deep learning framework based on rs-fMRI to classify patients with DOC with an accuracy of up to 0.86.^49^

### 5.3 Underlying Neuronal Mechanisms of Disorders of Consciousness

The neuronal correlates of consciousness (NCCs) are recognized as the minimal NCCs that are collectively necessary and sufficient to produce conscious experience.^50^ According to the mesocircuit model of recovery of consciousness,^3^ during normal cognitive processing, the central thalamus is regulated by the dominant corticothalamic feedback produced by the frontal area, and the activation of the central thalamus broadly derives activity of associative fronto-parietal, temporal, and occipital areas. It has been reported that DOC may be related to the disconnections in thalamo-cortical and long-range cortico-cortical pathways.

Corazzol et al.^51^ reported a UWS patient restoring consciousness after vagus nerve stimulation, and the results showed an increase in theta power over the right inferior parietal and parieto-temporal-occipital border, suggesting that vagus nerve stimulation enhances information communication within the centro-posterior network, leading to behavioral improvement. A positron emission tomography (PET) study reported that functional preservation in the left occipital region of DOC patients may reflect their perceptions of the external environment, whereas extensive functional preservation in the right cerebral hemisphere may reflect a motivation of a communicative nature.^52^ In addition, another similar study found that the more pronounced decrease in metabolic connectivity of the frontal, parietal, and temporal lobes with the occipital and limbic systems in HIE suggests that cognitive processes in these patients may not be interfacing with visual input.^53^ It has been found that cerebral metabolism or blood flow is in the normal range in patients in a VS.^54^ In this study, the significant difference in correlation between the MCS and UWS was also observed over the occipital cortex. Specifically, the correlation of L_OC was significantly higher in the UWS than that in the MCS. However, the underlying mechanisms of the phenomenon were unclear. The findings of this study suggested that the occipital cortex is one of the important brain regions associated with consciousness. However, what should be mentioned is that, given the limited sample size, further validation through multicenter, large-sample studies is needed. The differences observed in the current research may be attributed to the differences in sample characteristics, analytical methodologies, and neuroimaging techniques employed across different studies.^55^

### 5.4 Significance of Using rs-fNIRS to Evaluate Residual Consciousness

Spontaneous neural activity accounts for ∼95% of the total energy consumption of the brain, whereas only 5% is allocated to external task-related activity. This indicates that spontaneous neural activity may offer more extensive and profound information about brain function.^56^^,^^57^

In the past few years, numerous studies have identified stable resting-state functional connectivity (RSFC) patterns within several functional brain systems, including those responsible for motor control, visual, auditory, etc.^58^ Moreover, deficits in functional connectivity have been associated with several neurological and psychiatric disorders, revealing that the RSFC can be used as a valuable tool for evaluating brain functional activity.^59^^–^^61^ A comparative study has demonstrated that fNIRS can provide comparable RSFC measures to fMRI, thus producing direct evidence for the validity of optical brain connectivity and the optical brain network analysis in evaluating functional brain integration using resting-state data.^62^

In the RSFC studies, complex brain networks in diverse experimental modalities based on graph theory have further revealed the important topological properties of human brain networks, which are increasingly becoming a new hot spot in resting-state brain connectivity studies. The metrics of global efficiency ($E_{g}$) and characteristic path length ($L_{p}$) were used to quantify the overall transmission capacity of a network. An increased $E_{g}$ indicates that the network has a high capacity for parallel information transfer and that nodes are well-integrated. Meanwhile, a reduced $L_{p}$ implies that the network has a more compact structure, allowing for faster information transfer between any two nodes.^62^ The findings in this study showed that the MCS group exhibited higher global efficiency ($E_{g}$) and shorter characteristic path length ($L_{p}$) than the UWS group. This was in line with the previous study. For instance, several recent resting-state fNIRS studies have employed the $E_{g}$ index to evaluate the therapeutic efficacy of DBS. The research has demonstrated that effective DBS can enhance functional integrity and improve inter-brain communication. The $E_{g}$ index indicates a positive correlation between the changes in the $E_{g}$ index and the changes in the CRS-R index.^30^^,^^33^^,^^63^ Another study using $E_{g}$ found that MCS and UWS exhibited varying degrees of loss of topological architecture, which reduces the reliability and effectiveness of information transmission.^32^

Collectively, the results suggested that the global characteristics of the brain network could reflect the level of consciousness, and $E_{g}$ emerges as a promising functional biomarker for the quantification of residual consciousness in DOC patients.

We hope that the current study could provide useful insights into the evaluation of residual consciousness in patients with DOC. This study also confirmed that the global properties of the brain network have great potential to be used as a biomarker for distinguishing MCS from MCS.

### 5.5 Limitations and Further Consideration

First, the sample size is relatively limited. In subsequent exploratory studies, the enrollment criteria should be more carefully controlled, and the individual variability in gender, etiology, damaged brain areas, etc. should be considered. The findings of this pilot study should be further investigated and validated through multicenter studies with a larger aggregated sample size. Second, the resting-state data in this study were collected only once for each subject. Considering the frequent fluctuations in consciousness levels in this unique population, it is essential to conduct multiple assessments across different periods. Such repeated assessments are crucial for further validating the reliability and stability of the findings presented in this study. Third, in this study, PCA was used to eliminate the extracerebral contamination. In further explorative studies, short-channel fNIRS data should also be recorded to further improve the data quality. Moreover, improved data processing techniques and advanced experimental designs should be implemented to conduct a more thorough investigation of residual consciousness in patients with DOC. Despite these limitations, this study provided valuable insights into the assessment of residual consciousness for patients with DOC. We are confident that with the ongoing advancement in neuroimaging technology and medical care, patients with DOC will increasingly benefit in the near future.

## Data Availability

The datasets generated and/or analyzed during the current study are available from the corresponding author upon reasonable request.
